# Thrombotic complications of glucocorticoids and anabolic steroids

**DOI:** 10.1016/j.rpth.2025.103208

**Published:** 2025-10-07

**Authors:** Gerard Gurumurthy, Jecko Thachil

**Affiliations:** 1 The University of Manchester, Manchester, UK; 2 MAHSC Professor, The University of Manchester, Manchester, UK

**Keywords:** anabolic steroids, androgens, arterial thromboembolism, glucocorticoids, steroids, thrombosis, venous thromboembolism, VTE

## Abstract

Glucocorticoids and anabolic-androgenic steroids are used for medical and nonmedical purposes. There are thrombotic risks associated with both classes of steroids. Glucocorticoid therapy has been shown to increase the relative risk of venous thromboembolism. Glucocorticoids are also associated with arterial events. The mechanisms involve the exacerbation of cardiovascular risk factors and its effects on coagulation, platelet function, and endothelial integrity. Anabolic-androgenic steroids use similarly predisposes users to both venous thromboembolism and arterial thromboses. It is driven by androgen-induced polycythemia, platelet hyper-aggregability, procoagulant shifts in haemostatic proteins, and vascular injury. Preventive strategies involve minimizing steroid exposure. When thrombosis does occur, standard anticoagulant regimens are indicated. The preference is for low-molecular-weight heparin or direct oral anticoagulants to avoid warfarin-steroid interactions. The management of thrombotic events also involves re-evaluation of ongoing steroid need, close monitoring for bleeding, and multidisciplinary coordination. Current evidence largely derives from observational and pharmacologic studies. Nonetheless, clinicians must adopt a vigilant and individualized approach to balance the benefits of steroid therapy against its potential for serious thromboembolic complications.

## Essentials

• Glucocorticoids and anabolic-androgenic steroids increase venous and arterial thromboses.
• Preventive strategies involve minimizing steroid exposure, where possible.
• There is a paucity of data to guide anticoagulation management.
• Choice of anticoagulation should generally avoid warfarin-steroid interactions, where possible.

## 1 Introduction

Glucocorticoids and anabolic-androgenic steroids (AAS) are 2 distinct classes of steroid hormones widely used in medical and nonmedical contexts. Glucocorticoids, such as prednisolone and dexamethasone, are potent anti-inflammatory and immunosuppressive agents prescribed for conditions ranging from autoimmune diseases to cancer. AAS, which are synthetic derivatives of testosterone, are used clinically for hypogonadism or cachexia and, more commonly, misused as performance-enhancing drugs. An estimated 1 in 5 American adults received a short-term oral glucocorticoid in a 3-year period and 1% to 8% of gym-going individuals report using AAS [1,2].

Both classes of drugs have been implicated in thrombotic complications, which raises concerns for venous and arterial thromboses in the diverse patient populations who may receive them. Glucocorticoid therapy has long been recognized to contribute to cardiovascular risk factors such as hypertension, diabetes, and dyslipidaemia [3,4]. Emerging evidence indicates that exogenous corticosteroids independently can create a hypercoagulable state [5]. Similarly, AAS abuse has been linked to arterial events and venous thromboembolism (VTE) [6]. While early data on AAS-related thrombosis came mostly from case reports, recent large cohort studies have confirmed significantly elevated cardiovascular and thrombotic risk in AAS users [6,7]. Understanding the thrombotic complications of these drugs is of substantial clinical importance given the long-term risks and complications associated with thromboembolic diseases [8].

## 2 Glucocorticoids and thrombotic risk

Multiple epidemiologic studies have linked glucocorticoids to VTE. In a Danish population-based case-control study of 38 765 VTE cases, oral glucocorticoid use carried an odds ratio of 3.1 for a first VTE event, after adjusting for comorbidities [9]. The study included systemic glucocorticoids (betamethasone, methylprednisolone, prednisolone, prednisone, triamcinolone, and hydrocortisone) as well as inhaled and intestinal-acting glucocorticoids. In the systemic glucocorticoids cohort, the adjusted incidence risk ratio (IRR) for VTE were as follows: new users: 3.06 (95% CI: 2.77-3.38), continuing users: 2.02 (95% CI: 1.88-2.17), recent users: 1.18 (95% CI: 1.10-1.26), and former users: 0.94 (95% CI: 0.90-0.99). Recent analyses employing self-controlled case series and cohort designs have also provided high-level evidence that the thrombotic risk is at least partly attributable to glucocorticoid exposure itself. One study of 2547 patients found the IRR for first VTE during oral glucocorticoid treatment was 3.5 (95% CI: 2.6-4.8) compared with periods off treatment [3]. The authors used a self-controlled case series, added a 7-day preprescription window to capture flare-related risk, split exposure into 0-7, 8-30, 31-180, and >180 days after initiation, and lastly, assessed initial daily and 30-day cumulative prednisolone-equivalent doses as proxies for disease severity. Notably, VTE risk was highest in the early phase of therapy, where the IRR peaked at 5.3 in the first week of glucocorticoid use and remained about 2-3-fold elevated in the first few months. Part of this risk was apparent in the week prior to starting steroids (IRR = 2.5, 95% CI: 1.1-5.7), reflecting that acute disease flares requiring glucocorticoids also contribute to thrombosis. Nonetheless, the continued elevation during treatment and a decline after 6 months suggest a pharmacologic effect superimposed on baseline risk. In the same study, glucocorticoid therapy approximately doubled the hazard of recurrent VTE in patients with prior thrombosis (hazard ratio [HR] = 2.7, 95% CI: 1.6-4.8), suggesting that steroids can trigger new thrombi and recurrences. Interestingly, the risk of VTE is also observed in new users of intranasal corticosteroids (adjusted IRR = 2.2, 95% CI: 1.7-2.9) [9].

Beyond VTE, long-term glucocorticoid use has been associated with arterial thrombotic events, although disentangling steroid effects from confounding factors is challenging. Chronic steroid therapy often occurs in conditions that inherently increase cardiovascular risk, and steroids exacerbate traditional risk factors like hyperglycemia and dyslipidemia [3,4]. Epidemiologic studies have noted excess rates of myocardial infarction and stroke in patients on prolonged moderate-to-high dose glucocorticoids [[10], [11], [12], [13]], but quantifying the independent contribution of the drug is difficult. It is well established, however, that iatrogenic Cushing’s syndrome can accelerate atherosclerosis and arterial disease [5,14,15]. Thus, alongside VTE risk, clinicians should also be mindful of potential arterial events in patients on chronic steroids, especially in the presence of other cardiovascular risk factors.

The absolute risk increase conferred by glucocorticoids depends on the clinical context and dose-duration relationship. A database study of 1.5 million adults found that short courses (<30 days) of oral corticosteroids were associated with significantly elevated adverse event rates in the ensuing 90 days [1]. Within 30 days of steroid initiation, the incidence of VTE was over 3 times higher in steroid users than in non-users (IRR = 3.3, 95% CI: 2.7-4.0). This risk increase was evident even at modest prednisone-equivalent doses < 20 mg/day. Although the absolute VTE incidence in this generally low-risk outpatient cohort was small, the population impact is nontrivial given the frequency of steroid prescriptions.

## 3 AAS and thrombotic events

AAS use, whether in the form of high-dose testosterone therapies or as part of bodybuilding regimens, has emerged as an important risk factor for thrombosis and cardiovascular disease. Historically, evidence for AAS-related thrombosis came from case reports of young, otherwise healthy athletes developing unexpected thrombi. For instance, case reports have described pulmonary embolism or deep vein thrombosis in weightlifters taking oral androgens and cerebral infarctions in AAS users in young adults [16].

Until recently, robust epidemiologic data were scarce due to the clandestine nature of AAS recreational use. However, a large national cohort study has provided high-quality evidence quantifying the cardiovascular risk of AAS use. In this study, 1189 male AAS users identified through antidoping sanctions were followed over an average of 11 years [7]. Confounding was addressed by matching AAS users to 59 450 age-matched and sex-matched controls with synchronized cohort entry dates and analyzing incident outcomes using cause-specific Cox models that treated death as a competing risk. Models were further adjusted for baseline age, diabetes, educational length, and occupational status. Lifestyle factors and AAS dose/duration were not captured. The AAS users showed a markedly higher incidence of major thrombotic and cardiac events; adjusted HRs were 3.0 for acute myocardial infarction and 2.4 for VTE compared with non-users. HR for other cardiovascular outcomes were also elevated: 2.3 for arrhythmia, 3.6 for heart failure, and 8.9 for cardiomyopathy.

In the context of AAS abuse by athletes and recreational bodybuilders, the risk may be further amplified by the supratherapeutic doses and polypharmacy. The doping population often uses AAS cycles with weekly androgen doses in the gram range, far exceeding physiological replacement (>1g/week) [17,18]. They may also concurrently use erythropoietin, growth hormone, or stimulants, compounding thrombotic risk [17]. Small observational studies of weightlifters using AAS have documented incidences of VTE, acute coronary syndromes, and sudden cardiac death that exceed expected background rates [19,20].

The thrombotic risk of AAS is not limited to the excess doses used in bodybuilding; therapeutic testosterone supplementation in older men has been linked to thrombosis. A case-control study of nearly 1 000 000 patients, including over 39 000 testosterone users, found a significant increase in VTE incidence during the first 6 months of testosterone therapy in men without other VTE risk factors (IRR = 1.6, 95% CI: 1.1-2.4) [21].

## 4 Pathophysiology of glucocorticoids-induced thrombosis

**Figure 1:**
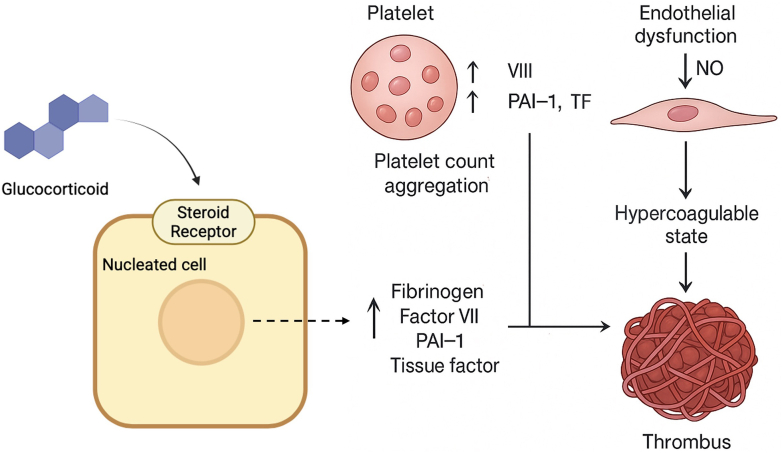
Mechanism of glucocorticoid-induced thrombosis. Glucocorticoid excess promotes thrombosis through increased procoagulant factors, impaired fibrinolysis, endothelial dysfunction and enhanced platelet activation.

Excess glucocorticoids, whether endogenous, as in Cushing’s syndrome, or exogenous, from therapy, may induce a prothrombotic state through multiple mechanisms (Figure 1). Haemostatic studies in patients with active Cushing’s syndrome provide a model for the changes seen with high-dose glucocorticoid exposure. Key coagulation and fibrinolytic parameters are shifted in a direction that favors thrombi formation and stability [5]. Specifically, glucocorticoid excess may lead to increased levels of procoagulant factors such as fibrinogen, factor VIII, and von Willebrand factor while simultaneously elevating levels of fibrinolysis inhibitors such as plasminogen activator inhibitor-1 and thrombin-activatable fibrinolysis inhibitor in patients with Cushing’s syndrome [22]. Functionally, these changes manifest as a shortened activated partial thromboplastin time and a prolonged clot lysis time in plasma-based assays [22,23]. The heightened coagulability of Cushing’s syndrome is reflected in an increased risk of VTE observed in this cohort. One meta-analysis determined that these individuals are at a 17- to 18-fold increased risk of VTE compared with the general population [23].

Glucocorticoids may also increase platelet count and reactivity, although data are mixed. Corticosteroids can cause a demargination of neutrophils and possibly platelets, and one study showed dexamethasone acutely increases circulating immature platelets and promotes thrombopoiesis in mice models [24]. *In vitro* studies of glucocorticoids-treated platelets showed a dose-dependent decrease of adenosine diphosphate–induced aggregation, TxB2 production, and arachidonic acid release from the platelet membrane [25]. In contrast, some *in vitro* data suggest very high glucocorticoid concentrations might inhibit platelet activation via thromboxane pathways [26]. Clinically, however, patients on steroids often exhibit thrombocytosis if there is an underlying inflammatory process [27]. Platelet function *in vivo* is likely enhanced by the proinflammatory cascade and cortisol’s permissive effects on catecholamines, rather than a direct steroid effect, but further research is warranted.

Endogenous cortisol excess is associated with hypertension and decreased endothelial nitric oxide due to oxidative stress, contributing to endothelial dysfunction associated with chronic use [28,29]. Additionally, glucocorticoids may increase blood viscosity by raising haematocrit and leukocyte counts [30,31].

It is also important to acknowledge the interplay between glucocorticoids and the underlying diseases they are prescribed for. Inflammatory disorders themselves drive prothrombotic changes. For instance, several inflammatory conditions elevate fibrinogen and von Willebrand factor levels as acute phase reactants [[32], [33], [34]]. Thus, the pathogenesis of steroid-related thrombosis in clinical practice likely involves a combination of the steroid’s direct haemostatic effects and indirect effects via disease modulation. Distinguishing these is challenging, but the net clinical observation is the observed heightened thrombotic risk in this cohort.

## 5 Pathophysiology of anabolic steroids–induced thrombosis

**Figure 2:**
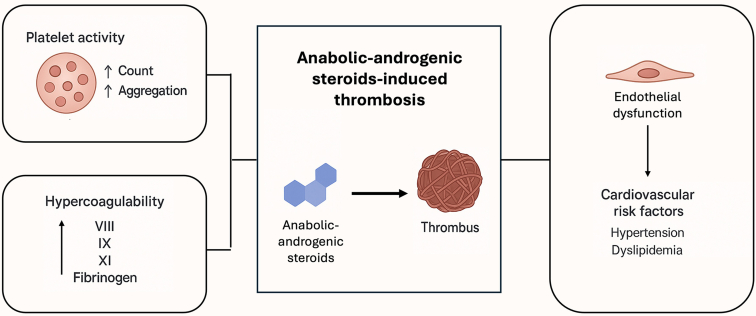
Mechanism of anabolic-androgenic steroid (AAS)-induced thrombosis. AAS enhances erythropoiesis, platelet aggregation via thromboxan A2 pathways and procoagulant shifts in coagulation proteins. It also induces vascular insult through dyslipidemia, hypertension and cardiomyopathy.

AAS influences thrombosis risk through a multifaceted impact on blood constituents and the vascular system (Figure 2). A hallmark effect of AAS and testosterone, in general, is stimulation of erythropoiesis. Androgens increase circulating red cell mass by promoting erythropoietin production and suppressing the hepcidin-mediated restriction on iron availability [[35], [36], [37]]. Clinically, this manifests as polycythemia in a significant fraction of AAS users, especially with injectable and high-dose regimens [36]. Polycythaemia increases blood viscosity and can predispose to stasis and thrombosis in peripheral veins [38]. Thus, the prothrombotic effect of AAS is partly driven by this erythrocytotic, hyper-viscous state akin to that seen in polycythemia vera where thrombotic events are a dominant cause of morbidity.

AAS use may also alter platelet function and the coagulation cascade. Androgens have been shown to enhance platelet aggregation through multiple mechanisms. One *in vitro* study found that testosterone exposure increased thromboxane A_2_ (TxA_2_) release from platelets and upregulated platelet TxA_2_ receptor density, thereby amplifying platelet aggregation responses [39]. In that study, peak platelet thromboxane generation occurred about 4 weeks into testosterone administration, mirroring the timeframe when clinical thrombotic events have been observed in some new users. Another study comparing testosterone effects in men and women noted that exogenous testosterone led to increased platelet aggregation in response to adenosine diphosphate and reduced platelet nitric oxide, but only in male subjects [40]. The cumulative evidence suggests that androgens promote a proaggregatory platelet phenotype through increased cyclooxygenase activity and TxA_2_ synthesis, heightened platelet-activating factor levels, and decreased antiaggregatory nitric oxide within platelets.

The effects of AAS on the coagulation cascade have also been investigated but findings are somewhat conflicting. Several studies have reported that AAS users exhibited elevated levels of certain clotting factors such as prothrombin, factor (F)IX, and FVIII [41,42]. However, not all studies confirmed these procoagulant changes [43,44]. The recent health risks of anabolic-androgenic steroid use by male amateur athletes (HAARLEM) prospective study followed 100 men through a self-administered AAS cycle and measured serial changes in haemostatic parameters [6]. During active AAS use (median 13-week cycles at high doses), several changes in the levels of multiple coagulation proteins were noted in participants, including Protein S (22%, 95% CI: 15-29) D-dimer (1.3-fold, 95% CI: 1.2-1.5), and clot lysis time (8 minutes longer, 95% CI: 5-10). FVIII and von Willebrand factor levels remained unchanged. Notably, all abnormal changes reversed back to baseline within 3 months after discontinuation of AAS.

Thrombin-generation studies around AAS-induced thrombosis vary by population and timing. In a cross-sectional study of current and former AAS abusers, calibrated automated thrombography showed 15% higher endogenous thrombin potential alongside higher prothrombin and FX [41]. This is consistent with a net procoagulant shift that may persist after cessation. In contrast, the prospective HAARLEM cohort measured coagulation repeatedly through real-world AAS cycles and found broad rises in several coagulation proteins and D-dimer, but a modest reduction in endogenous thrombin potential during use and reversal after discontinuation [6]. Finally, one study noted a decreased fibrin clot lysis time in current and former AAS abusers [45]. These findings nonetheless suggest that AAS abuse may be associated with increased thrombotic risk.

AAS may also affect the cardiovascular system which indirectly facilitates arterial thrombus formation. AAS use has been associated with a marked reduction in high-density lipoprotein cholesterol and an increase in low-density lipoprotein, thus accelerating atherosclerotic plaque development [46]. Endothelial dysfunction is also a consequence of AAS abuse, with studies associating impaired vasodilatory responses and increased oxidative stress in AAS users, contributing to an atherogenic and prothrombotic endothelium [47]. Additionally, AAS use has been associated with hypertension, left ventricular hypertrophy, and dilated cardiomyopathy with heart failure [7], which increases the risk for arterial thrombi [48,49].

In summary, AAS create a prothrombotic environment through [1] increased red cell mass and blood viscosity [2], enhanced platelet aggregability via thromboxane and other pathways [3], a shift in coagulation/fibrinolysis balance toward clot stability (e.g. high plasminogen activator inhibitor-1, prolonged clot lysis), and [4] vascular injury from dyslipidemia, hypertension, and cardiomyopathy leading to conditions favoring thrombosis. Ongoing research is needed to elucidate the true changes of coagulation parameters and its effect on thromboembolic risk.

## 6 Prophylaxis and prevention of thrombotic complications

**Figure 3:**
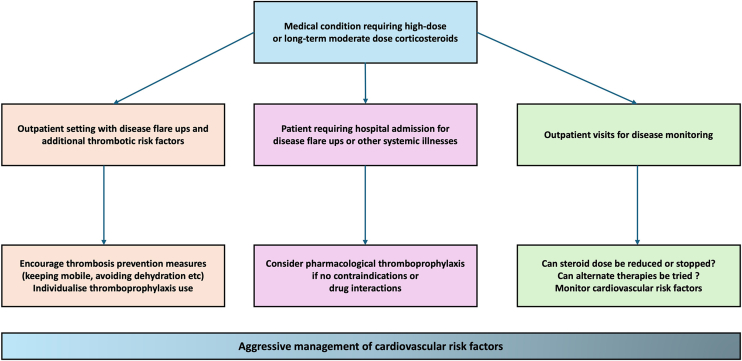
Preventive strategies for steroid-associated thrombosis. Key measures include minimizing steroid dose adn duration and managing comorbid cardiovascular risk factors. Thromboprophylaxis may be considered in certain cases with its use highly individualized.

Preventive strategies for steroid-associated thrombosis revolve around mitigating risk factors and judicious use of thromboprophylaxis in high-risk scenarios (Figure 3). Given that steroids may be medically necessary, the goal is to balance the benefits of therapy with measures that minimize thrombotic risk. These measures differ somewhat between glucocorticoids and anabolic steroids, but there are common principles of risk stratification, risk factor modification, and targeted use of anticoagulant prophylaxis.

### 6.1 Glucocorticoid therapy: risk mitigation and thromboprophylaxis

The first principle in preventing glucocorticoid-related thrombosis is to use the lowest effective dose and shortest duration of steroid therapy necessary for disease control. Studies have shown a clear dose-response effect against VTE risk [1]. Clinicians should therefore regularly re-evaluate the ongoing need for glucocorticoids and employ steroid-sparing therapies when possible to reduce cumulative exposure. In patients with chronic inflammatory diseases requiring long-term steroids, aggressive management of the underlying disease to enable tapering off steroids will inherently reduce thrombotic risk over time.

There are no formal guidelines that mandate primary VTE prophylaxis solely for glucocorticoid use in the outpatient setting. The decision to use an anticoagulant in this context should be individualized, considering the patient’s thrombotic risk factors against the risk of disease status and bleeding.

However, clinicians may implement prophylaxis for inpatient application of glucocorticoid. Most hospitalized individuals with acute illness should receive pharmacologic VTE prophylaxis per standard protocols, and the addition of glucocorticoids further justifies that approach. In the case of Cushing’s syndrome or patients on comparably high steroid doses, several centers often provide prophylactic anticoagulation. A recent survey of European reference centers for Cushing’s syndrome found that majority of centers (31 of 42) routinely administer thromboprophylaxis to patients with active Cushing’s [50]. The majority of centers restricted prophylaxis to the inpatient setting, but a subset also prescribed prophylactic anticoagulation in the ambulatory setting for severe cases. Factors influencing the decision to start prophylaxis included the severity of hypercortisolism and any history of VTE. Only one center had a standardized protocol, reflecting variability and the lack of high-level evidence to guide thromboprophylaxis in this setting.

### 6.2 Anabolic steroids: prevention in medical and nonmedical contexts

For AAS, the most effective prevention of thrombosis is avoidance of nonmedical use. Primary prevention focuses on education and cessation of abuse [51]. Health professionals who encounter patients using AAS should counsel on the significant thrombotic and cardiac risks and strongly encourage discontinuation of steroid use. Cessation of AAS is associated with normalization of many risk factors within months [6,52], thereby substantially reducing ongoing thromboembolic risk. Clinicians may recognize AAS abuse through biochemical abnormalities, including very low serum high-density cholesterol and sex hormone-binding globulin concentrations and unexplained erythrocytosis [52]. In athletes, engaging experts in sports medicine and psychology may aid in addressing the dependency and body image issues that fuel continued AAS abuse [51].

In scenarios where AAS or high-dose androgens are medically necessary, risk mitigation is key rather than discontinuation. There is a lack of evidence to guide the best approach to this. However, clinicians should consider several factors that are associated with thromboembolic risk. Monitoring and managing erythrocytosis, for example, is crucial. Clinical guidelines for testosterone therapy recommend checking haematocrit levels periodically; if haematocrit rises above a threshold (typically >50% to 54%) [53,54], action should be taken. The Endocrine Society Clinical Guidelines recommend either discontinuation of AAS or phlebotomy to reduce haematocrit [54].

Addressing modifiable co-risk factors is another preventive strategy. Encouraging smoking cessation in a patient on testosterone can mitigate an interactive effect that greatly increases polycythemia and thrombosis risk [55]. Controlling blood pressure and cholesterol in older men on testosterone will help reduce the acceleration of atherosclerosis.

Pharmacologic prophylaxis in AAS users is not routinely prescribed in the way it might be for high-risk glucocorticoid patients, mainly because AAS use outside of medical indications is often for recreational purposes. Again, there are no formal guidelines to guide thromboprophylaxis in these situations, and the decision to anticoagulate must be individualized.

## 7 Treating thrombosis secondary to steroids

**Figure 4:**
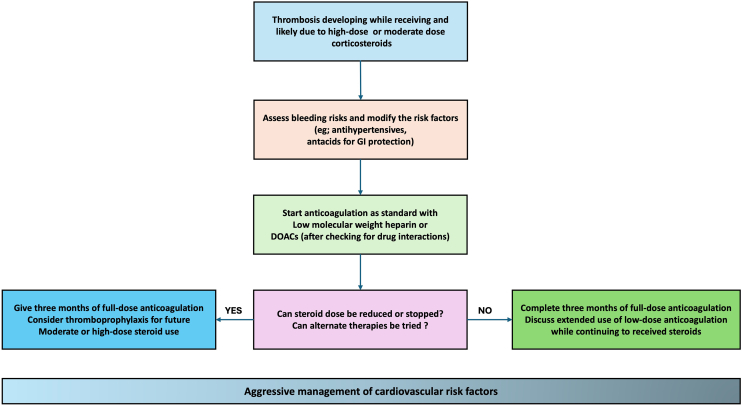
Management of steroid associated thrombotic events. Treatment involves standard anticoagulation (preferably low-molecular weight heparin or direct oral anticoagulants to avoid warfarin-steroid interactions). Reassessing ongoing steroid therapy and monitoring for bleeding and renal function are crucial.

When thrombotic events occur in the context of steroid use, standard principles of anticoagulation and cardiovascular care apply, but several special considerations arise (Figure 4). Management must address not only the acute treatment of the thrombus but also decisions about continuing or modifying the steroid therapy, choosing an appropriate anticoagulant regimen mindful of drug interactions, and balancing bleeding risks that may be heightened by the steroid itself.

### 7.1 Choice of anticoagulant—treating the acute thrombotic event and thromboprophylaxis

Selecting an anticoagulant for a patient on steroids requires consideration of potential drug-drug interactions and patient-specific factors. Concomitant glucocorticoid therapy can potentiate warfarin’s effect, leading to elevated international normalized ratio (INR) and bleeding risk. A retrospective study found that starting an oral corticosteroid in a patient on stable warfarin caused the INR to rise above therapeutic range in 62% of cases, with a mean INR increase of 1.24 within about 1 week [56]. Thus, if warfarin is used in a patient on glucocorticoids, close INR monitoring and dose adjustments are imperative during the first 1 to 2 weeks of cotherapy. AAS is also known to potentiate warfarin’s anticoagulant effect. Pharmaceutical labels warn against the concomitant use of both drugs due to an increased risk of bleeding. The dose of warfarin had to be reduced by 80% to 85% to maintain the desired INR [57].

Direct oral anticoagulants (DOACs) have the advantage of fixed dosing and fewer interactions. They are increasingly first-line for treating VTE. However, one must consider that certain steroids can alter DOAC metabolism. Dexamethasone, for instance, is a moderate inducer of CYP3A4 and P-glycoprotein [58]; in patients on long-term dexamethasone, levels of DOACs that are CYP3A4 substrates (rivaroxaban and apixaban) might be modestly reduced [59,60]. In most cases, the interaction is not strong enough to contraindicate DOAC use, but careful observation for efficacy should be considered. Glucocorticoids also can cause changes in renal function [61], which is relevant because DOAC doses depend on renal clearance. Ensuring the patient’s renal function is adequate for the chosen DOAC is standard but particularly important if steroids have caused fluctuating oedema or blood pressure that might affect kidney function.

A thrombotic event linked to steroid use should be managed according to existing guidelines for that type of thrombosis. There is no evidence that steroid-associated thrombi require different anticoagulant dosing; they should be treated with full-intensity anticoagulation just as any provoked VTE. Given the paucity of data to guide optimum management of acute thrombotic events in this group, it may be that if the patient’s thrombus is associated with a transient steroid exposure, the event may be considered provoked and a finite duration of anticoagulation (typically 3 to 6 months) is considered, provided the provoking factor is not ongoing. On the other hand, if the patient must remain on steroids long-term, or continues AAS use, one might treat the event as a persistent-risk situation and consider extended anticoagulation beyond 6 months, depending on bleeding risk. For arterial thromboses in steroid users, management follows cardiology or neurology standards (e.g. aspirin, P2Y12 inhibitors, thrombolysis if indicated acutely, etc.), but with attention to removing or reducing the steroid risk factor to prevent recurrence.

### 7.2 Bleeding risk and complications

Balancing bleeding risk is a universal challenge in anticoagulation. Steroid use adds specific concerns. Glucocorticoids, for example, may cause mucosal ulceration in the gastrointestinal tract [62]. Similarly, long-term steroid users might have steroid-induced peptic ulcers or gastritis in certain individuals [63]. Adding an anticoagulant or antiplatelet in that setting increases the risk of gastrointestinal bleeding. Prophylactic use of proton pump inhibitors can be considered in patients on combination of steroids and anticoagulants, especially if other risk factors for gastrointestinal bleed exist (e.g. prior ulcer, non-steroidal anti-inflammatory drugs use) [64]. Hence, the threshold to initiate prophylactic anticoagulation in a steroid-treated patient must account for bleeding risk factors. Multidisciplinary input can be helpful for complex cases to tailor prophylaxis safely.

## 8 Conclusion

While steroids may remain medically invaluable, their thrombotic risks are a significant consideration. Both glucocorticoid and anabolic steroid use can provoke a hypercoagulable state via distinct but sometimes overlapping mechanisms, leading to VTE and arterial events. Clinicians must be proactive in mitigating these risks through judicious use of steroids, appropriate use of thromboprophylaxis, and careful management of any thrombotic events that do occur. For now, a careful, evidence-informed, and patient-tailored approach is our best tool to prevent and manage the thrombotic complications of glucocorticoid and anabolic steroid use.
